# Differential contributions of an antimicrobial effector from *Verticillium dahliae* to virulence and tomato microbiota assembly across natural soils

**DOI:** 10.1186/s40168-026-02376-y

**Published:** 2026-03-16

**Authors:** Wilko Punt, Anton Kraege, Sabine Metzger, Natalie Schmitz, Jinyi Zhu, Stéphane Hacquard, Michael Bonkowski, Nick C. Snelders, Bart P. H. J. Thomma

**Affiliations:** 1 Institute for Plant Sciences, Cluster of Excellence on Plant Sciences (CEPLAS), University of Cologne, Cologne, 50674 Germany; 2 Max Planck Institute for Plant Breeding Research, Cluster of Excellence on Plant Sciences (CEPLAS), Cologne, 50829 Germany; 3 Institute for Zoology, Cluster of Excellence on Plant Sciences (CEPLAS), University of Cologne, Cologne, 50674 Germany

## Abstract

**Background:**

Throughout their life cycle, plants associate with diverse and complex microbial communities collectively known as their microbiota. These microbiota contribute to plant performance and health by enhancing nutrient acquisition, modulating immunity, and providing a microbial barrier against pathogens. To successfully colonize their hosts, pathogens must overcome not only plant immune defenses but also this microbial barrier. For example, the soil-borne fungal pathogen *Verticillium dahliae* secretes the antimicrobial effector Ave1 to suppress antagonistic microbes and facilitate infection. Although many plant pathogens, including *V. dahliae*, inhabit both plant-associated and soil environments, how antimicrobial effectors contribute to pathogen establishment across these diverse ecological contexts remains poorly understood.

**Results:**

To explore this question, we assembled a collection of natural soils differing in physicochemical properties and microbiota composition. Using three host plant species—barley, tomato, and cotton—we found that root-associated bacterial and fungal communities were primarily shaped by type of soil, whereas phyllosphere microbiota were mainly determined by plant species identity. On tomato, we further observed that the effector Ave1 differentially contributed to *V. dahliae* virulence depending on the soil of origin. While Ave1 consistently altered tomato-associated microbiota across all soils tested, the specific microbial taxa affected varied between soils.

**Conclusions:**

Our findings demonstrate that the impact of the antimicrobial effector Ave1 on microbiota composition and pathogen virulence is context-dependent, influenced by the specific soil-derived microbial community that assembles on the host. This work highlights the ecological complexity of effector functions and suggests that pathogen success in natural environments depends on dynamic interactions with both the plant host and its microbiota.

Video Abstract

**Supplementary Information:**

The online version contains supplementary material available at 10.1186/s40168-026-02376-y.

## Introduction

Plants host diverse microbial communities, known as the plant microbiota, which mainly include bacteria, fungi, and protists [55]. These microorganisms colonize all plant parts, and together with the host plant, form a unified biological entity often referred to as the holobiont [57]. Apart from seed-borne microbes inherited from the mother plant in the previous plant generation, the majority of microbes that make up the plant microbiota are recruited from environmental niches. While some microbes are transmitted through the air, the surrounding soil serves as the primary reservoir from which plants acquire most of their microbiota [10]. Soil properties such as pH, nutrient availability, organic carbon content, temperature and redox status shape the pool of microbes available for recruitment into the plant microbiota [17]. Consequently, the physicochemical properties of soil have a strong influence on plant microbiota assembly, as evidenced by the distinct microbial communities found in plants grown on different soils [4, 53]. At the same time, host genetics exert selective pressure on which taxa colonize and persist in the plant microbiota [4, 31, 58]. This is particularly evident in the formation of the core microbiota, which is a consistent subset of microbial taxa that reliably establish within the microbiota of a plant, even when plants are grown in diverse soils [1, 31].

To date, numerous studies have separately demonstrated the importance of the bulk soil on the one hand, and of host genetics on the other hand, in structuring plant microbiota [4, 18, 31, 45, 53, 54, 58, 59]. These studies have examined plant-associated microbes in diverse natural environments, where abiotic factors like local climate and weather can influence microbiota assembly, or have compared microbiota of different plant species grown in the same soil at a single location [39, 58, 59]. However, studies that simultaneously evaluate the contribution of plant genetics and differential bulk soil microbiota on plant microbiota assembly, for instance by using various plant species in diverse natural soils while controlling for environmental influences, remain scarce [15, 54].

Microbes that establish in the plant microbiota interact with the host plant in various ways. Many microbes interact with plants as neutral commensals, while other microbes can be beneficial to the plant, or can be pathogenic and cause disease [23]. The community balance and composition of the microbiota plays an important role in plant health and performance, particularly by contributing to defense against pathogens [14]. Notably, plants have the ability to actively recruit beneficial microbes in response to pathogen attack. For instance, cucumber plants infected by the soil-borne pathogen *Fusarium oxysporum* f. sp. *cucumerinum* recruit *Bacillus amyloliquefaciens* to reduce disease severity [29]. Over longer timescales, such plant-driven recruitment of beneficial microbes can result in the formation of disease-suppressive soils, where susceptible plants can grow in the presence of pathogens without experiencing severe disease symptoms [14]. A well-documented example, is the response of wheat plants to infection by *Gaeumannomyces graminis* var. *tritici*, the causal agent of “take-all” disease In this case, wheat recruits beneficial *Pseudomonas* species that antagonize the pathogen through the secretion of antimicrobial compounds, ultimately contributing to disease suppression over successive planting cycles in particular fields [43, 52]. Importantly, protection via microbial recruitment is not limited to direct antagonism of pathogens. Some beneficial microbes enhance plant immunity through the induction of systemic defense responses [41]. For example, *Arabidopsis thaliana* plants infected with the foliar pathogen *Hyaloperonospora arabidopsidis* (Hpa) selectively promote the growth of three bacterial species in the rhizosphere. This recruitment boosts systemic resistance to Hpa, improves overall plant growth, and can even benefit subsequent plant generations by fostering a protective microbiome [3]. In this way, the plant microbiota has also often been considered as an additional layer of the immune system against pathogens by both inducing immune responses and directly antagonizing pathogens [5, 14, 16, 34].

While colonizing their hosts, plant pathogens secrete so-called effector molecules to promote host colonization by manipulating host physiology, including immunity [12, 24]. Recently, several studies have demonstrated that pathogens exploit effector proteins that possess antimicrobial activity to manipulate the host microbiota, and thus facilitate colonization [7, 8, 20, 26, 28, 35, 40, 47–49]. For example, the soil-borne fungal plant pathogen *Verticillium dahliae* exploits the antimicrobial effector protein Ave1 to suppress antagonistic Sphingomonadales bacteria during host colonization of tomato and cotton plants [49]. Interestingly, predictions from a machine learning tool suggest that 349 secreted *V. dahliae* effectors possess antimicrobial activity, indicating that *V. dahliae* may devote a substantial proportion of its secreted proteins to microbiota manipulation [36].

Fungal pathogens such as *V. dahliae* occupy a range of ecologically distinct niches throughout their life cycle [19, 21]. While they infect host plants during specific life stages, many also persist outside the host for extended periods, particularly in the soil [19, 25]. Soil microbial communities are generally more diverse than those associated with plants and vary substantially depending on the physicochemical properties of the soil [17, 51]. Accordingly, many pathogens are exposed to diverse microbial environments and must interact with a wide range of microbial taxa over time [50]. This is particularly relevant for broad host range pathogens like *V. dahliae*, which are adapted to numerous hosts and habitats and are thought to rely on antimicrobial effectors that facilitate interactions with different microbial communities [50, 55]. Building on previous studies that explored antimicrobial effector functions using a single type of soil [49], we hypothesize that the virulence contribution of antimicrobial effectors like Ave1, as well as their impact on microbial communities, may vary depending on the host-associated microbiota, which is largely derived from the bulk soil microbial community.

Here we report the establishment of a collection of natural soils that are diverse in both physicochemical characteristics and microbiota composition. We use this resource to simultaneously assess the contributions of the diverse types of soil and the plant genotype to plant microbiota assembly under controlled greenhouse conditions by analyzing fungal and bacterial communities associated with barley, cotton and tomato plants grown on each soil. Additionally, we utilize the soil collection to investigate the impact of the antimicrobial effector protein Ave1 on tomato microbiota composition and its role in *V. dahliae* virulence during infection of tomato plants harboring distinct microbiota.

## Results

### Composing a collection of diverse natural soil samples

**Fig. 1:**
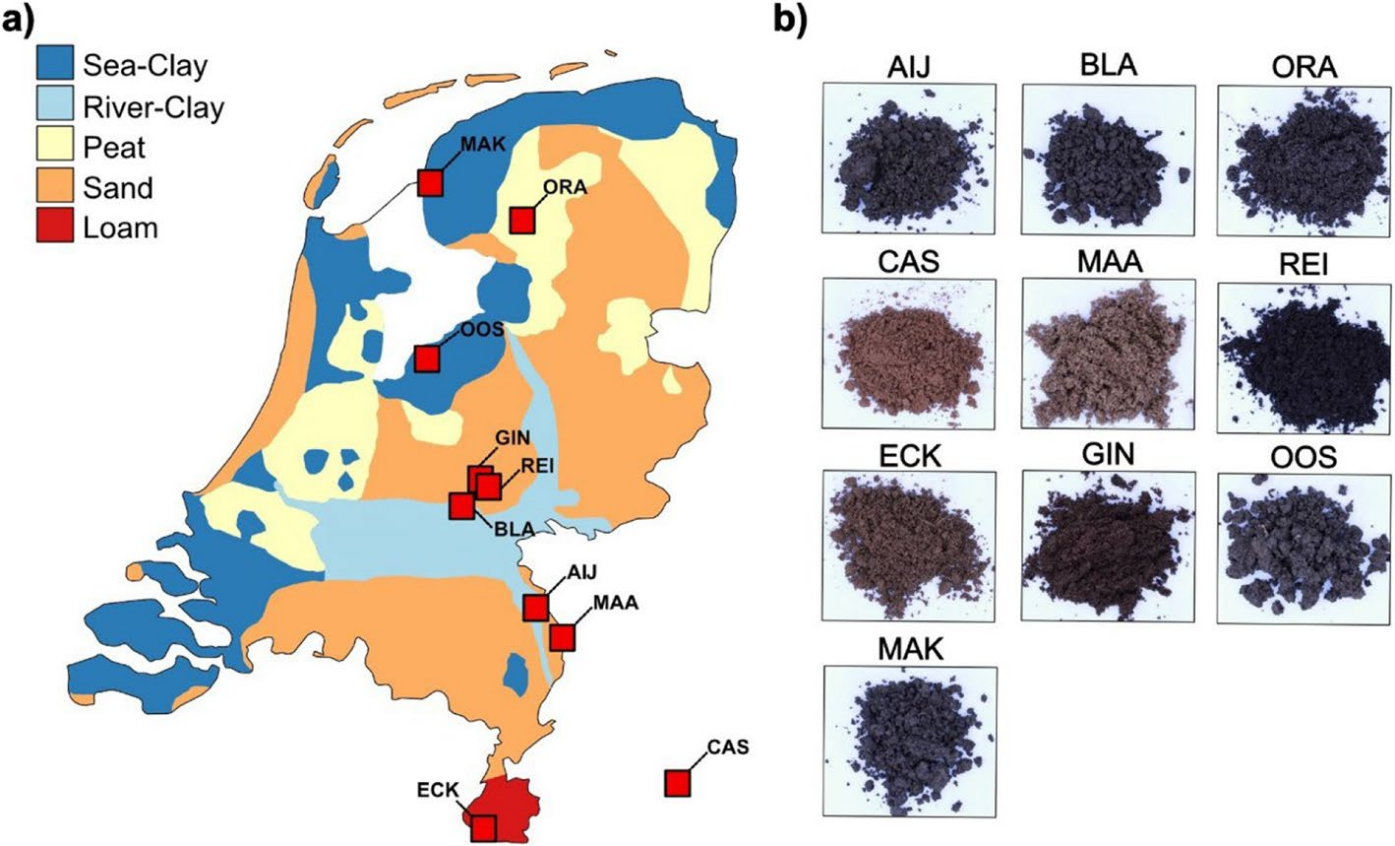
Establishment of a natural soil collection. **a** Soil collection sites in the Netherlands. The map is colored according to major types of soil in the Netherlands. Sampling locations are indicated by red squares. **b** Pictures of each soil from the soil sample collection

To study microbiota assemblies and the role of antimicrobial effector proteins of fungal plant pathogens in diverse soils we composed a collection of natural soil samples. We collected our soil samples in the Netherlands given the well-documented types of soil and the opportunity to sample a wide range of distinct types of soil on a relatively short geographical distance ([22]; Fig. 1a). In total we collected samples from nine different natural soils which map onto five major conventional soil classes: river clay, sea clay, sand, peat and loam (Suppl. Table 1). Sampling sites were selected to avoid agricultural usage. In order to eliminate weeds and the majority of roots, the top 10 cm of soil was removed and the subsequent 30 cm of soil was collected. Besides the nine Dutch soils, we included the well-characterized and intensively studied Cologne agricultural soil [4].

**Fig. 2:**
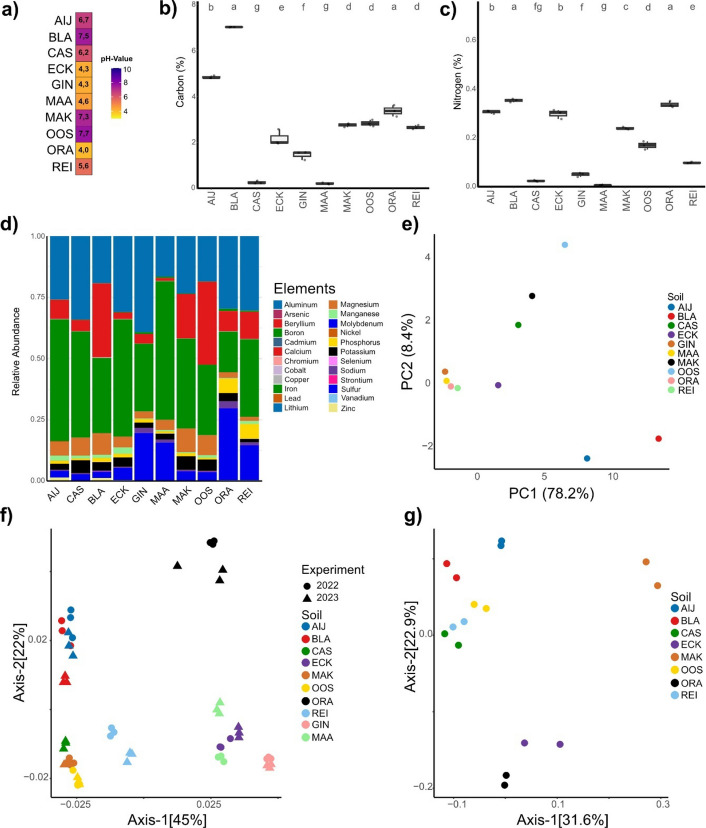
Physicochemical and microbiota analysis of the natural soil collection. **a** Heatmap of pH-values. **b** Boxplots displaying soil carbon contents. Different letters indicate statistical differences based on One-Way-Anova (Tukey HSD-Test pval < 0.05). **c** Boxplots displaying soil nitrogen contents. Different letters indicate statistical differences based on One-Way-Anova (Tukey HSD-Test pval < 0.05). **d** Relative abundance barplot for elements measured with ICP-MS. **e** Principal component analysis (PCA) of the elemental profiles measured with ICP-MS. **f** Principal coordinate analysis (PCoA) using weighted unifrac distances displaying bacterial bulk soil microbiota. Datapoints are shaped according to collection timepoint. PERMANOVA significance test showed significant clustering by type of soil (*P* < 0.001) but not by year (*P* = 0.6732). **g** PCoA using weighted unifrac distances displaying fungal bulk soil microbiota

The diversity of our soil sample collection is apparent from visible differences in soil texture and appearance (Fig. 1b). To determine differences in physicochemical properties of our soil samples, we measured pH, the amount of total organic carbon and nitrogen, as well as element levels for all soil samples. The sandy soils (sand, peat, loam) displayed relatively low pH values, between 4.0 and 5.6, while the clay soils (river clay, sea clay) displayed higher pH values ranging from 6.2 to 7.7 (Fig. 2a). With respect to carbon content, particularly the two river clay soils collected in Aijen (AIJ) and Blauwe Kamer (BLA) stood out with the highest carbon content of 4,83% and 7,01% respectively. The lowest carbon content was measured for the Cologne agricultural soil (CAS) with 0,26% and the sand soil collected in Maasduinen (MAA) with 0,21% (Fig. 2b). A similar pattern was observed for the nitrogen content, as the highest value was measured for the river clay BLA with 0,35%, while lowest values were again determined for MAA at 0,006% and CAS at 0,02% (Fig. 2C). Further, we also performed a total element analysis by conducting a HNO_3_-based element extraction followed by inductively coupled plasma mass spectrometry (ICP-MS) measurement. The elemental profiles of our soil samples were dominated by iron, calcium and aluminum (Fig. 2d). Notably, when computing a principal component analysis (PCA) of the ICP-MS elemental raw data we observed separation according to types of soil, as the clay soils separated from the sandy soils and the CAS-soil (Fig. 2e).

Many of the physicochemical properties are known to influence soil microbiota composition [17]. To determine the bulk soil microbiota, we conducted 16S amplicon sequencing and analyzed the β-diversity by computing a principal coordinate analysis (PCoA) using the weighted Unifrac distance (Fig. 2f). As expected, we observed separation of the microbiota according to the type of soil. Notably, we observed that apart from Reijerscamp (REI) the sandy soils collected from de Ginkelse Heide (GIN), Maasduinen (MAA), Oranjewoud (ORA) and ECK separate from the clay soil samples.

To investigate the consistency of the bulk soil microbiota, we compared the bulk soil microbiota of soil samples that were collected in two consecutive years; 2022 and 2023. In the PCoA, soils collected in the different years clustered, demonstrating a high degree of stability of these natural bulk soil microbiota (Fig. 2f). PERMANOVA significance testing showed significant clustering by types of soil (*P* < 0.001) but not by year (*P* = 0.6732). Additionally, we sequenced the soil from the same sampling sites two years later in 2025, displaying the same clustering by types of soil (Suppl. Figure 1). Collectively, our data characterize the diversity of our natural soil sample collection with respect to physicochemical properties and bulk soil microbiota.

### Drivers of bacterial community assembly in roots and phyllosphere microbiota

Several studies have demonstrated that the soil as well as plant genetics influence plant microbiota assemblies [4, 18, 31, 45, 53, 54, 58, 59]. These investigations typically involved plants collected from diverse natural environments, where microbiota assembly may additionally be affected by various abiotic factors, such as local climate and weather conditions, or they involve different plant species grown in the same soil at the same site [39, 58, 59]. However, studies that simultaneously assess the contributions of different soils and of the plant genetics to microbiota assembly by examining diverse plant species grown in diverse natural soils while eliminating the impact of environmental factors remain scarce. Thus, we used our soil sample collection to investigate how plant-associated microbiota assemble across different plant species when grown under controlled conditions in a greenhouse. Specifically, we grew tomato (*Solanum lycopersicum*), cotton (*Gossypium hirsutum*), and barley (*Hordeum vulgare*) on the ten soils of our soil sample collection.

We first assessed how the diverse properties of the natural soils influence plant growth, by measuring plant canopy areas at three weeks after sowing. Cotton, tomato and barley plants grew on all soils except on the GIN and MAA soil samples, while tomato additionally failed to grow on ECK. Significant growth differences were observed across soils for each plant species (Suppl. Figure 2). Generally, the highest plant growth was observed on clay soil. For cotton the highest plant growth was determined on the MAK soil samples with an average canopy area of 39,76 cm^2^. Barley and tomato plants displayed highest plant growth on the BLA soil with barley plants reaching an average canopy area of 10,89 cm^2^ and tomato 22,23 cm^2^. Lowest plant growth for all three plants species was observed on the ORA soil, with average canopy areas of 23,44 cm^2^ for cotton, 1,8 cm^2^ for tomato plants and 1,42 cm^2^ for barley plants (Suppl. Figure 2). These results highlight the influence of the different soils on plant growth.

**Fig. 3:**
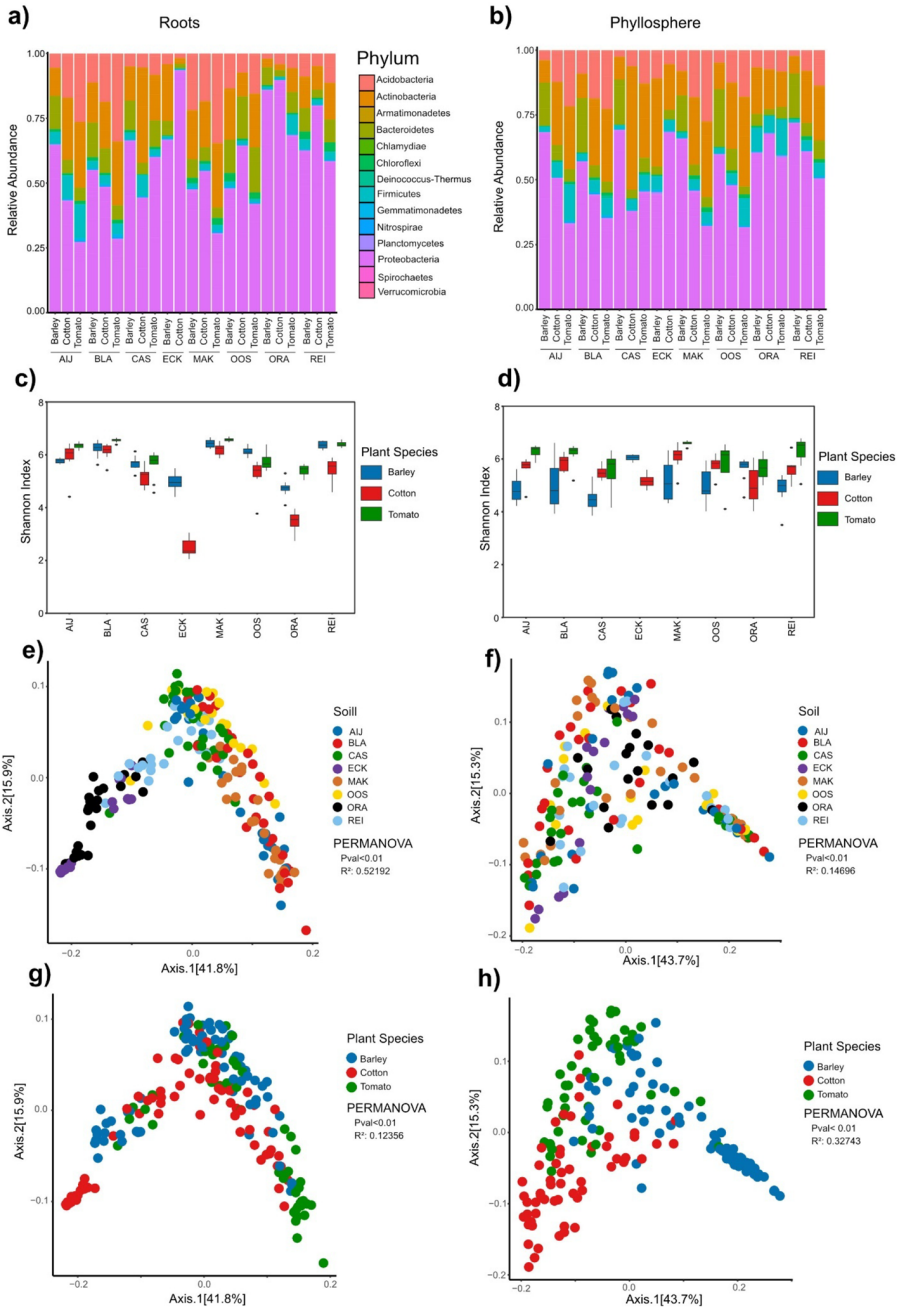
Bacterial composition of root and phyllosphere associated microbiota of barley, cotton and tomato plants grown on the different natural soils. **a** Relative abundance in percentage on phylum level of the bacterial root microbiota. **b** Relative abundance in percentage on phylum level of the bacterial phyllosphere microbiota. **c** Shannon index of root microbiota. **d** Shannon index of phyllosphere microbiota. **e** Principal coordinate analysis (PCoA) based on weighted Unifrac distance of root microbiota. Datapoints are colored according to type of soil. **f** PCoA based on weighted Unifrac distance of phyllosphere. Datapoints are colored according to type of soil. **g** PCoA based on weighted Unifrac distance of root microbiota. Datapoints are colored according to plant species. **h** PCoA based on weighted Unifrac distance of phyllosphere microbiota. All PERMANOVAs are performed with 9999 permutations. Datapoints are colored according to plant species

Next, we assessed the bacterial root and phyllosphere microbiota of the diverse plants grown on the soil collection by performing 16S rRNA sequencing. Bacterial communities in the root-associated microbiota were dominated by Proteobacteria, Actinobacteria, Acidobacteria, and Bacteroidetes across all soils and plant species. Notably, we observed considerable variation among individual plants of the same species grown in the same soil, despite prior homogenization. This may result from heterogeneity that persists in the natural soils samples even after mixing (Suppl. Figure 3). Nevertheless, as expected, we observed strong differences in bacterial community composition between plant species grown on the same soils. For instance, on the river clay soil AIJ, over 50% of the bacterial community in the barley root microbiota consisted of Proteobacteria, compared to only 25% of Proteobacteria in the tomato root microbiota. Rather, the tomato root microbiota on AIJ harbored higher proportions of Acidobacteria and Actinobacteria (Fig. 3a). To assess the diversity of the root-associated microbial communities, we investigated microbial alpha diversities by calculating the Shannon index for each bacterial community sample. Notably, Shannon indices for the root microbiota varied across plant species and soils, with no plant species consistently exhibiting higher or lower diversity compared to the other species across the soils (Fig. 3c). This was also supported by the calculated Hill numbers (Suppl. Figure 7). The lowest Shannon index was measured for cotton plants grown on ECK (2,49), whereas the most diverse communities were assembled by tomato plants grown on MAK (6,57).

To further disentangle the contributions of the type of soil and plant species to microbial diversity in the root microbiota, we analyzed β-diversities by conducting Principal Coordinate Analyses (PCoAs) based on weighted UniFrac distances. In the root-associated microbiota, bacterial communities grouped primarily according to the type of soil, with sandy soils (ORA, ECK, REI) separating from clay soils (AIJ, BLA, OOS, MAK, CAS). The type of soil accounted for 52,2% of the observed variation within the microbiota, suggesting a dominant contribution to shaping root-associated bacterial communities (Fig. 3e). We observed a “horseshoe effect”, which is thought to arise when applying linear ordination such as PCoA to gradient data [37]. Therefore, we additionally calculated nonlinear ordination-based NMDS plots, which show a similar pattern of our data as the PCoA analysis (Suppl. Figure 8). Although also plant species significantly contributed to root-associated microbiota differentiation, it explained only 12,4% of the variation (Fig. 3g; Suppl. Figure 4b). Thus, root-associated microbiota are primarily structured according to the type of soil, and furthermore by plant species.

Next, we assessed if the patterns observed for root microbiota similarly hold true for phyllosphere microbiota. Similar to root microbiota, phyllosphere microbiota were dominated by Proteobacteria, Actinobacteria, Acidobacteria, and Bacteroidetes across all soils and plant species (Fig. 3b). Also, for the phyllosphere microbiota we observed considerably variation between individual plants of the same species when grown in the same soil (Suppl. Figure 4). Notably, we observed strong differences between phyllosphere microbiota of different plant species grown in the same soil. Interestingly, these differences were similar across soils. For example, the tomato phyllosphere microbiota consistently exhibiting the lowest levels of Acidobacteria, followed by cotton and then barley in seven of the eight soils tested, with ECK as exception (Fig. 3b). Next, we assessed community diversity in the phyllosphere microbiota by calculating Shannon indices (Fig. 3d). In the phyllosphere, the lowest Shannon indices were determined for barley plants grown on CAS (4,51) and AIJ (4,84), whereas highest values were again observed for tomato plants grown on MAK (6,59) and REI (6,27). Notably, the alpha diversity of bacterial phyllosphere microbiota displayed a more structured pattern when compared with the alpha diversity in the root microbiota, as barley consistently exhibited the lowest alpha diversity across six out of the eight soil samples, followed by cotton and then tomato (Fig. 3d). This suggests that the plant species has a more pronounced influence on community diversity in the phyllosphere microbiota when compared with root-associated microbiota. We also analyzed β-diversities by conducting Principal Coordinate Analyses (PCoAs) based on the weighted UniFrac distances of the phyllosphere microbiota. Like the root-associated microbiota, the phyllosphere microbiota exhibited significant separation based on the type of soil, albeit that this explained substantially less variation (14,7%). Rather, plant species was the strongest determinant of the phyllosphere community composition, accounting for approximately 32,7% of the observed variation (Fig. 3f, Suppl. Figure 3).

Collectively, our findings indicate that the soil is the strongest driver of bacterial microbiota diversity in the root microbiota, while plant species plays a more significant role in shaping bacterial phyllosphere communities.

### Drivers of fungal community assembly in root-associated and phyllosphere microbiota

To assess whether patterns observed for bacterial microbiota across plant species grown on our soil collection also apply to the fungal component of the microbiota, we conducted ITS sequencing. First, we examined the fungal communities in the bulk soil microbiota of the eight soil samples used for the plant microbiota assembly study. This analysis revealed that the sand-like soils ECK and ORA separate from the clay soil. The REI soil, although also a sandy-soil, grouped with the clays. This indicates that the soil sample collection harbors distinct fungal communities (Fig. 2g).

**Fig. 4:**
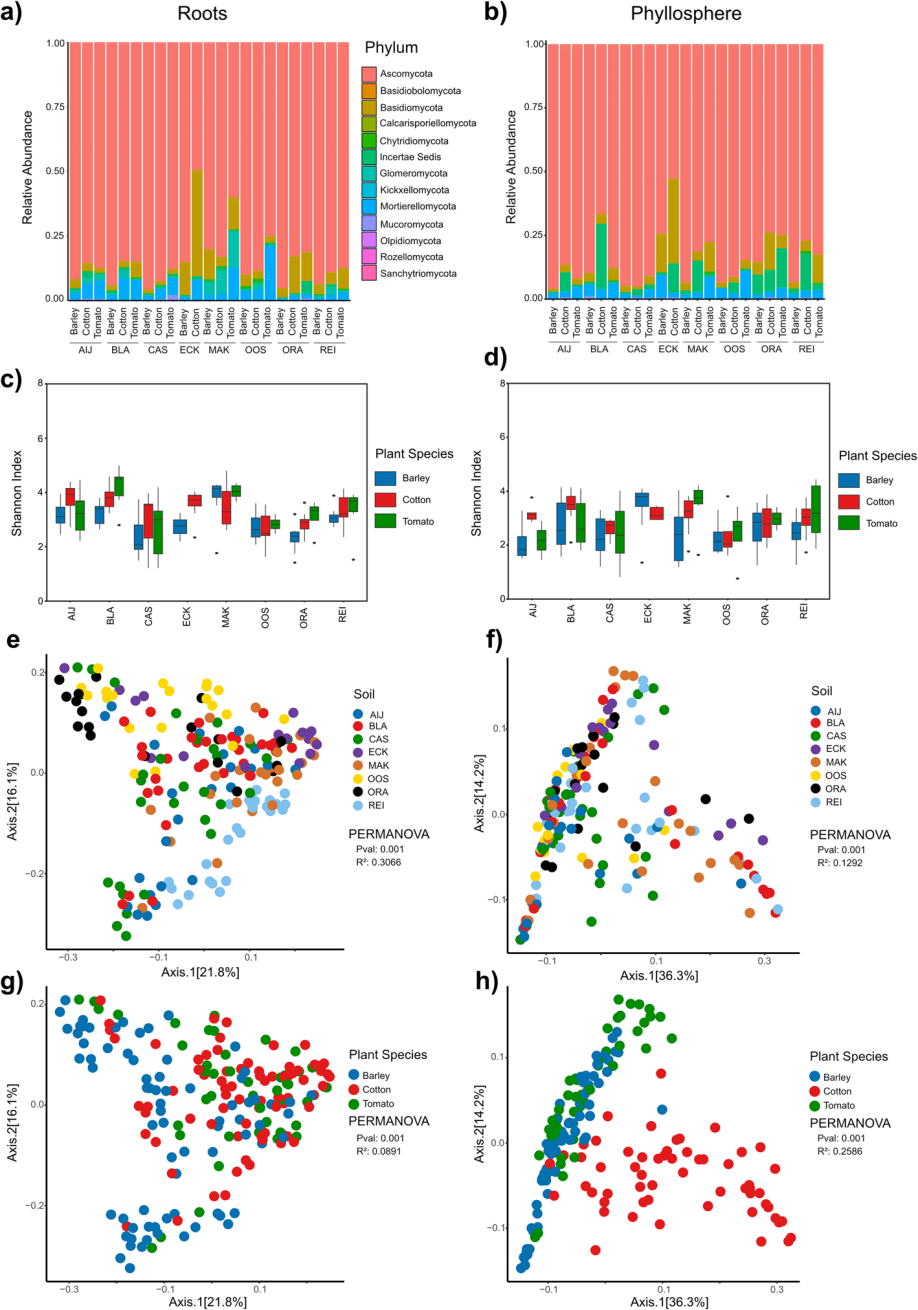
Composition of the fungal root and phyllosphere associated microbiota of barley, cotton and tomato plants grown on the different natural soils. **a** Relative abundance in percentage on phylum level of the fungal root microbiota **b** Relative abundance in percentage on phylum level of the fungal phyllosphere microbiota **c** Shannon index of root microbiota. **d** Shannon index of phyllosphere microbiota. **e** Principal coordinates analysis based on weighted Unifrac distance of root microbiota. Datapoints are colored according to type of soil. **f** PCoA based on weighted Unifrac distance of phyllosphere. Datapoints are colored according to type of soil. **g** PCoA based on weighted Unifrac distance of root microbiota. Datapoints are colored according to plant species. **h** PCoA based on weighted Unifrac distance of phyllosphere microbiota. Datapoints are colored according to plant species. All PERMANOVAs are performed with 9,999 permutations

Analysis of the fungal communities in the root-associated microbiota revealed that fungal communities across plant species and type of soil were dominated by fungal species from the phyla Ascomycota, with Basidiomycota and Mortierellomycota (Fig. 4a). Notably, the fungal composition of the root microbiota is also influenced by plant species across soil samples. For instance, on ECK, the fungal communities in the barley root microbiota contained more than 80% Ascomycota, while the fungal root microbiota of cotton plants contained only 50% Ascomycota, with a substantially higher abundance of Basidiomycota (Fig. 4a; Suppl. Figure 6). Shannon index calculations revealed lower alpha diversities of the root-associated fungal communities when compared with bacterial communities, with no consistent patterns of alpha diversity based on the plant species emerging across soil samples. Analysis of the β-diversity by performing a PCoA using the weighted Unifrac distance matrix revealed that root-associated fungal communities separate based on the soil sample in which the plants were grown, explaining 31% of the variation observed in the fungal microbiota (Fig. 4e). Root-associated fungal communities also displayed weak separation according to plant species, which explained 9% of the variation (Fig. 4g; Suppl. Figure 6b).

Overall, these findings suggest that fungal communities in the root-associated microbiota are primarily shaped by the type of soil. As expected, also in the phyllosphere microbiota the fungal communities were dominated by Ascomycetes, followed by Basidiomycetes and Mortierellomycetes (Fig. 4b; Suppl. Figure 5). Similar as for the alpha diversity in the root-associated fungal microbiome we did not observe any alpha diversity patterns based on plant species or the type of soil in the fungal phyllosphere microbiota (Fig. 4d). The β-diversity analysis of the fungal community in the phyllosphere microbiota revealed weak separation based on the type of soil, which explained 13% of the variation (Fig. 4f). Notably, similar as for the bacterial phyllosphere microbiota, we observed strong separation of the fungal phyllosphere community based on plant species, which explained 26% of the variation (Fig. 4h; Suppl. Figure 5b). Collectively, our dataset reveals that fungal communities in the root-associated microbiota are more strongly influenced by types of soilthan by plant species, while the plant species acts as the primary driving factor for fungal communities in the phyllosphere microbiota.

### Differential contribution of antimicrobial effectors to fungal virulence across types of soil

**Fig. 5:**
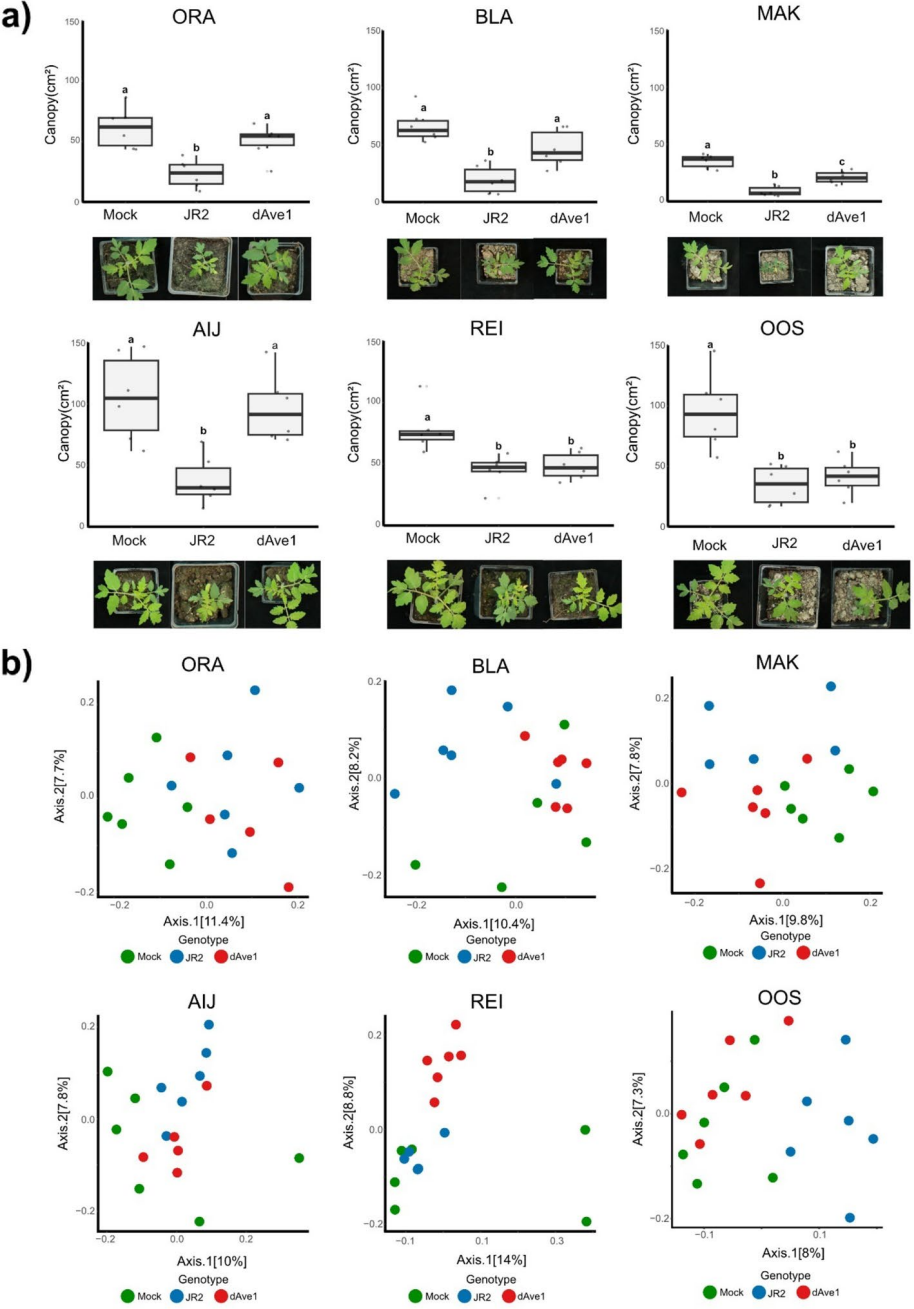
Antimicrobial effector Ave1 differentially contributes to virulence of *Verticillium dahliae* depending on the soil. **a** Canopy area in cm^2^ of tomato plants grown on the different natural soils at 14 dpi with mock treatment, wild-type *V. dahliae* (JR2) or an *Ave1* deletion mutant (dAve1). Different letters indicate statistical differences based on One-Way-Anova (Tukey HSD-Test pval < 0.05). Pictures display a representative plant per treatment. **b** Principal coordinate analysis (PCoA) based on Unifrac distances of the root microbiota of tomato plants grown on different soils at 14 dpi with wild-type *V. dahliae* (JR2) or an *Ave1* deletion mutant (dAve1)

The plant microbiota plays an important role in plant health, fitness and defense against plant pathogens [55]. To colonize their hosts, plant pathogens have evolved antimicrobial effector proteins to manipulate host-associated microbiota [35]. For instance, *V. dahliae* uses the antimicrobial effector Ave1 to suppress antagonistic microbes during host colonization. Ave1 was demonstrated to facilitate host colonization of cotton and tomato plants grown in potting soil through targeting, amongst others, antagonistic Sphingomonadales bacteria [49]. As a globally distributed soil-borne pathogen with a broad host range, *Verticillium dahliae* successfully colonizes host plants across diverse types of soil, which likely harbor distinct microbial communities [27, 46]. We hypothesized that the outcome of effector-mediated microbiota manipulation may vary depending on the host-associated microbiota, which is largely assembled from the surrounding bulk soil microbiota. To address this, we assessed the virulence contribution of the antimicrobial effector Ave1 by growing plants on our soil collection and inoculating them with either wild-type *V. dahliae* or an *Ave1* deletion mutant [13, 49]. As we have studied the interaction of *Verticillium dahliae* with tomato most intensively, and the Ave1 effectors acts as a major virulence factor on this plant species [13, 49], we focused our experiment on tomato plants [13, 49]. We observed a significant reduction in biomass of tomato plants inoculated with the wild type strain when compared with plants inoculated with the *Ave1* deletion strain on AIJ, BLA, ORA and MAK, indicating that Ave1 contributes to fungal virulence on these soils. In contrast, no such difference was observed for plants grown in OOS and REI, suggesting that Ave1 differentially contributes to fungal virulence across soils (Fig. 5a). To rule out the possibility that the observed differences between soil types were due to coinfections with naturally occurring *Verticillium* strains, we quantified the relative abundance of *Verticillium* in bulk soil and found only low abundances with no significant differences (Suppl. Figure 9). Previous work demonstrated that Ave1 also negatively impacts the abundance of other taxa, including Verrucomicrobiales, Chitinophagaceae, Flavobacteriales and Burkholderiales during infections of cotton and tomato plants grown on potting soil [49]. We then asked whether variation in the abundance of these bacteria in the root-associated microbiota of tomato plants could explain the differences in virulence contribution of Ave1 across soils. To test this, we measured their relative abundance in tomato plants grown in the different natural soil samples. Of the tested taxa, Sphingomonadales, Flavobacteriales, and Burkholderiales showed no significant differences in relative abundances across soils. While significant variation in relative abundance was observed for the Verrucomicrobiales and Chitinophagaceae on several soils, these differences did not correlate with the observed Ave1-related virulence phenotype (Suppl. Figure 10a).

To assess the impact of Ave1 on the tomato root-associated microbiota we investigated the microbiota composition of tomato plants that were mock-inoculated, or inoculated with *V. dahliae* strain JR2 or the *Ave1* deletion mutant. By computing PCoAs based on UniFrac distances, we observe differences in the microbiota composition of tomato plants across treatments on our soil collection (Fig. 5b). We further computed a PCoA based on UniFrac distances, focusing only on plants that were inoculated with *V. dahliae* and observed that the tomato microbiota from plants inoculated with the wild type and the deletion mutant consistently separated across all soils, except for the ORA soil (Suppl. Figure 11). Notably, we also observe such separation in the microbiota of plants grown on REI and OOS, even though we did not detect a virulence contribution of Ave1.

To investigate the bacterial taxa affected by Ave1 on the natural soils we conducted differential abundance analysis using DeSeq2 (v1.36.0; [30]) between the microbiota of plants inoculated with *V. dahliae* strain JR2 or the *Ave1*-deletion mutant. This analysis revealed significant shifts in microbiota composition at the genus level across all soil samples, including OOS and REI, even though no virulence contribution of Ave1 was observed on these soils (Suppl. Figure 10b). Notably, on each of the soils the effector causes distinct shifts in the microbiota (Suppl. Figure 10b). Collectively, our data indicates that the outcome of effector-mediated microbiota manipulation by *V. dahliae* is determined by the composition of the host-associated microbiota which, in turn, is influenced by the surrounding soil.

## Discussion

Plant microbiota contribute substantially to plant productivity, in part by serving as an additional barrier against invading pathogens [14, 35]. Over recent years, it has become evident that plant pathogens manipulate host microbiota through the secretion of antimicrobial effector proteins in turn, thus facilitating niche establishment and host colonization [7, 8, 20, 26, 28, 35, 40, 47–49]. Notably, many pathogens spend parts of their life cycles outside their hosts, where they encounter diverse microbial communities. However, how antimicrobial effectors aid fungal establishment across these diverse environments is still poorly understood. Here, we present a collection of natural soils that we thoroughly characterized in terms of their physicochemical properties as well as their microbiota compositions. Using this soil collection, we reveal that the antimicrobial effector protein Ave1 from soil-borne fungal plant pathogen *Verticillium dahliae,* which was previously demonstrated to facilitate host colonization through the suppression of antagonistic Sphingomonadales bacteria [49]*,* contributes to fungal virulence on tomato plants only in a subset of these soils. Our finding suggests that the virulence contribution of this effector is determined by the soil on which the host plant grows. Interestingly, differential virulence contributions have similarly been reported for another antimicrobial effector from *V. dahliae*, called Av2. While initially no contribution to fungal virulence was recorded [9], a subsequent study using a different growth substrate, likely with a distinct microbiota, revealed that Av2 interfered with the host plant’s ‘cry for help’ recruitment of beneficial *Pseudomonas* bacteria, leading to a clear virulence contribution of the effector [28]. These differences in virulence contributions of antimicrobial effectors are likely due to variation in soil microbiota, which impacts the composition of plant-associated microbial communities encountered by the pathogen during infection in turn. Interestingly, our microbiota analyses revealed that the Ave1 effector significantly altered the tomato microbiota on all tested soils. This implies that microbiota manipulation by the effector does not necessarily translate into measurable contributions to fungal virulence and thus, that this effector does not solely target antagonists of *V. dahliae* growth. We therefore infer that the presence or absence of antagonistic microbes that can be impacted by an antimicrobial effector will determine whether that effector contributes to fungal virulence during host infection. This hypothesis is supported by observations made for the *V. dahliae* antimicrobial effector protein Ave1L2. A previous study investigating Ave1L2 demonstrated that in communities artificially depleted of antagonistic Actinobacteria, described as a crucial target of the effector, the protein still impacted community composition albeit without a measurable virulence contribution [47].

Notably, the observed impact that Ave1 caused on the plant microbiota substantially differed across soils. Many antimicrobial effector proteins do not specifically act on a single antagonistic microbe, but rather act on multiple plant microbiota members, thus exerting broader, system-level impacts on microbial communities [8, 28, 48, 49]. Since plant microbiota function as networks of interdependent species [56], changes affecting one member can cascade through the community. Thus, removal or suppression of particular microbes by fungal effectors may trigger cascading shifts in community structure and function due to these intermicrobial interactions. This interconnectedness implies that the effects of antimicrobial effector activity on the microbiota can vary substantially between microbial communities, driven by the unique web of intermicrobial interactions in each environment.

Our study additionally provides a controlled comparison of how both types of soil and plant genotype influence microbiota assembly across different plant compartments. While previous studies have independently demonstrated that rhizosphere communities are heavily influenced by soil and phyllosphere communities by host genotype [4, 18, 31, 45, 53, 54, 58, 59] these insights were often derived from field studies conducted in divergent natural environments, where additional abiotic factors such as climate and weather may influence microbiota composition, or from experiments that varied either soil or plant species, but rarely both. Tkacz et al. [54] assessed microbiota assembly across four plant species grown in two distinct soils and demonstrated that soil has a stronger influence than plant species on shaping rhizosphere microbiota. In our study, we extend these findings by using a different set of plant species and a broader collection of ten diverse, well-characterized soils, including the Cologne agricultural soil [2, 4] and Reijerscamp soil [3, 42] under highly controlled greenhouse conditions. We not only confirm that the type of soil plays a dominant role in rhizosphere microbiota assembly, but also show simultaneously that, in contrast, phyllosphere communities are more strongly shaped by plant species rather than the type of soil. Notably, this work demonstrates a stronger degree of plant-driven selection on fungal communities in the phyllosphere compared to the rhizosphere, highlighting a pronounced filtering of microbes from the root to the shoot. This process is reminiscent of the “rhizosphere effect” previously described for bacteria along the soil–root gradient. Such selection effects represent an important aspect of microbiome assembly that remains underexplored in fungal communities relative to bacteria.

Taken together, our findings support the view that antimicrobial effector proteins are context-dependent components of fungal secretomes, rather than universally acting virulence factors with consistent effects across environments. Notably, a recent machine-learning analysis predicted that, for several fungi, at least one-third of effector proteins possess antimicrobial activity [36], suggesting that fungi may deploy large repertoires of such antimicrobial effectors to establish themselves in diverse environments. Deeper insight into their functions and the mechanisms underlying this environmental variability will not only advance our understanding of fungal niche adaptation but may also inform the development of more robust, microbiota-based disease control strategies for agriculture.

## Materials and methods

### Soil collection and storage

Natural soils were collected. Two soil collections were performed, in February 2022 and February 2023 at nine sites in the Netherlands: Makkum (53°05′09.8"N 5°26′20.3"E), Oranjewoud (52°57′11.7"N 5°57′45.6"E), Ginkelse Heide (52°02′10.7"N 5°43′38.9"E), Eckelrade (50°47′57.7"N 5°44′42.5"E) Maasduinen (51°28′34.3"N 6°11′34.9"E), Oostvaardersplassen (52°27′50.0"N 5°25′10.8"E), Reijerscamp (52°00′37.7"N 5°46′25.0"E), Blauwe Kamer (51°56′34.4"N 5°37′12.9"), Aijen (51°34′55.0"N 6°02′27.3"E). For collection, the top 10 cm of soil was removed and the subsequent 30 cm of soil was collected. After collection, soil samples were homogenized and rocks and pieces of plant material were removed before the soil was stored in sealed buckets at 8℃ until further use. Further, Cologne agricultural soil (50°57′27.8"N 6°51′22.4"E; [2]) was included.

### Physicochemical soil analysis

For physicochemical analysis, 50 g of each soil was freeze dried and ground to fine powder using a mortar and pestle. To measure soil pH, ground soil powder was suspended with 150 ml of distilled water and incubated for 1 h. Subsequently the pH was measured using a pH-electrode (Meddler Toledo, Giessen, Germany). Carbon and nitrogen levels were measured using the FLASH2000 CHNS/O analyzer (Thermo Fisher Scientific, Waltham, USA). To measure elemental contents, 100 mg of soil powder was weighed into metal-free centrifugation tubes (VWR, Radnor, USA). Samples were the soaked in 500 µl of 30% nitric acid for 2 h. Subsequently, the volume was adjusted to 1 ml with 30% nitric acid and the sample was incubated for 14 h at 65℃. Next, the suspension was incubated at 95℃ for 90 min. Samples were cooled to room temperature and 200 µl of hydrogen peroxide were added. Subsequently, the samples were incubated at 95℃ for 30 min. Next, the samples were diluted to 10 ml using MQ-water and centrifuged at 13,000 rpm for 1 h at 4℃. The supernatant was transferred to a clean metal-free 50 ml centrifugation tube and incubated at 4℃ overnight, followed by centrifugation at 13,000 rpm for 1 h at 4℃. Finally, 600 µl of supernatant were mixed with 2,4 ml of 2% nitric acid. ICP-MS measurements were carried out on an Agilent 7700 ICP-MS (Agilent Technologies, Waldbronn, Germany) in the Biocenter MS-Platform of the University of Cologne. All measurements were performed in technical triplicates and strictly followed the manufacturer`s instructions using He in the collision cell mode to minimize spectral interference.

### Plant growth assays

Tomato (*Solanum lycopersicum L.*) cultivar MoneyMaker, barley (*Hordeum vulgare*) cultivar GoldenPromise and cotton (*Gossypium hirsutum*) cultivar DDHY642201-AC were used for all assays. Before sowing, seeds were surface-sterilized using chlorine gas generated by adding 3 mL of hydrochloric acid (HCl) to 100 mL of bleach (sodium hypochlorite) in a 250 mL beaker placed inside a glass container sealed with a lid and parafilm and incubated for 5 h. After sterilization, the container was vented in a fume hood overnight. Subsequently, surface sterilized seeds were sown on soil and grown for three weeks in a greenhouse chamber with 16 h of light at 23 °C, followed by 8 h in darkness at 22°C. Plant growth was assessed by calculating canopy areas, for tomato and cotton based on overhead pictures and for barley plants based on side pictures using ImageJ [44]. Subsequently, plants were harvested for microbiota analysis. Tomato and cotton phyllosphere samples were collected by harvesting the stem from the soil-line to the cotyledons, while barley phyllosphere samples were collected by harvesting the first 5 cm of plant tissue above the soil line. To collect root microbiota samples, plants were uprooted and loose soil was removed from the root system through gentle shaking.

### Microbiota sequencing

Samples were manually ground to fine powder using a mortar and pestle. Subsequently, 400 mg of tissue or soil were used for DNA extraction using the DNeasy PowerSoil Pro Kit (Qiagen, Venlo, The Netherlands). Next, DNA was further purified using the Monarch PCR&DNA Clean Up kit (New England Biolabs, Ipswich, USA). DNA purity and concentration were assessed using the Qubit 4 fluorometer (Thermo Fisher Scientific, Waltham, USA) and the Nanodrop 2000 spectrophotometer (Thermo Fisher Scientific, Waltham, USA). DNA was used for the amplification of the variable regions 3–4 of the 16S region using primers 341f (ACTCCTACGGGAGGCAGCAG) and 806r (GGACTACHVGGGTWTCTAAT) in the presence of the mPNA (GGCAAGTGTTCTTCGGA) and pPNA (GGCTCAACCCTGGACAG) blocking clamps (PNABio, Newbury Park, USA). Additionally, amplification of the ITS2 region was conducted using the primers ITS3 (GCATCGATGAAGAACGCAGC) and ITS4 (TCCTCCGCTTATTGATATGC) in the presence of the ITS2 PNA (CGAGGGCACGTCTGCCTGG) blocking clamp (PNABio, Newbury Park, USA). All amplicons were sequenced on an Illumina MiSeq Platform (BGI-Genomics, Shenzhen, China). For the bulk soil microbiota from the soil collections, the V5-V7 regions were amplified with primers 799F (AACMGGATTAGATACCCKG) and 1139R (ACGTCATCCCCACCTTCC) and amplicons were similarly sequenced on an Illumina Miseq Platform (Cologne Center for Genomics, Cologne, Germany). Only samples with at least 10.000 reads were considered for the analysis. Data analysis was conducted as described previously [6, 49].

### Microbiota analysis

Sequencing data were processed using R v.4.2.0. as described previously [6, 49]. In brief, reads were demultiplexed with cutadapt (v4.1; [32]), then trimmed and filtered to an average paired read length of 412 bp with a Phred score of 30. OTUs were inferred from the trimmed reads using the DADA2 method (v 1.24; [6]). Taxonomy was assigned using the Ribosomal Database Project (RDP,v 18; [11]). The pyloseq package (v1.40.0; [33]) was used to calculate α- and β-diversity, while PERMANOVA was conducted with the vegan package (v2.6–4; [38]) package. Differential abundance analysis was done using the DESeq2 package (v1.36.0; [30]) for each type of soil individually using a negative binomial Wald test and a significance P adjusted threshold < 0.05.

### *Verticillium* inoculation assays

*Verticillium dahliae* inoculations were conducted on 10-day-old tomato plants. Inoculum was prepared by harvesting conidiospores of 10-day-old cultures of *V. dahliae* strain JR2 and an *Ave1* deletion mutant [13, 49] on potato dextrose agar (PDA; Carl Roth, Karlsruhe, Germany). The collected conidiospores were washed three times in MQ water, each time followed by centrifugation at 10.000 rpm for 10 min. Subsequently, the conidiospores were counted using a Neubauer chamber and the inoculum concentration was adjusted to 10^6^ conidiospores/ml. For the inoculations, plants were uprooted and the roots were rinsed with MQ-water before being placed into the conidiospore suspension for 8 min. Subsequently, plants were planted back into the soil. Disease symptoms were monitored at 14 dpi by measuring the tomato canopy area based on overhead pictures using ImageJ [44].

## Supplementary Information

Supplementary Material 1.

## Data Availability

16S profiling data are available in the NCBI Genbank database under BioProject PRJEB95937.

## References

[1] Almario J, Mahmoudi M, Kroll S, Agler M, Placzek A, Mari A, et al. The leaf microbiome of Arabidopsis displays reproducible dynamics and patterns throughout the growing season. MBio. 2022;13(3):e0282521. 10.1128/mbio.02825-21. 35420486 10.1128/mbio.02825-21 PMC9239250

[2] Bai Y, Müller DB, Srinivas G, Garrido-Oter R, Potthoff E, Rott M, et al. Functional overlap of the Arabidopsis leaf and root microbiota. Nature. 2015;528(7582):364–9. 10.1038/NATURE16192. 26633631 10.1038/nature16192

[3] Berendsen RL, Vismans G, Yu K, Song Y, de Jonge R, Burgman WP, et al. Disease-induced assemblage of a plant-beneficial bacterial consortium. ISME J. 2018;12(6):1496–507. 10.1038/s41396-018-0093-1. 29520025 10.1038/s41396-018-0093-1 PMC5956071

[4] Bulgarelli D, Rott M, Schlaeppi K, Ver Loren Themaat E, Ahmadinejad N, Assenza F, et al. Revealing structure and assembly cues for Arabidopsis root-inhabiting bacterial microbiota. Nature. 2012;488(7409):91–5. 10.1038/nature11336. 22859207 10.1038/nature11336

[5] Carrión VJ, Perez-Jaramillo J, Cordovez V, Tracanna V, de Hollander M, Ruiz-Buck D, et al. Pathogen-induced activation of disease-suppressive functions in the endophytic root microbiome. Science. 2019;366(6465):606–12. 10.1126/science.aaw9285. 31672892 10.1126/science.aaw9285

[6] Callahan BJ, McMurdie PJ, Rosen MJ, Han AW, Johnson AJA, Holmes SP. DADA2: high-resolution sample inference from Illumina amplicon data. Nat Methods. 2016;13(7):581–3. 10.1038/NMETH.3869. 27214047 10.1038/nmeth.3869 PMC4927377

[7] Chang H, Noel ZA, Chilvers MI. A β-lactamase gene of *Fusarium oxysporum* alters the rhizosphere microbiota of soybean. Plant J. 2021;106(6):1588–604. 10.1111/tpj.15257. 33788336 10.1111/tpj.15257

[8] Chavarro-Carrero EA, Snelders NC, Torres DE, Kraege A, López-Moral A, Petti GC, et al. The soil-borne white root rot pathogen *Rosellinia necatrix* expresses antimicrobial proteins during host colonization. PLoS Pathog. 2024;20(1):e1011866. 10.1371/journal.ppat.1011866. 38236788 10.1371/journal.ppat.1011866 PMC10796067

[9] Chavarro-Carrero EA, Vermeulen JP, Torres D, Usami T, Schouten HJ, Bai Y, et al. Comparative genomics reveals the in planta-secreted Verticillium dahliae Av2 effector protein recognized in tomato plants that carry the V2 resistance locus. Environ Microbiol. 2021;23(4):1941–58. 10.1111/1462-2920.15288. 33078534 10.1111/1462-2920.15288 PMC8246953

[10] Chialva M, Lanfranco L, Bonfante P. The plant microbiota: composition, functions, and engineering. Curr Opin Biotechnol. 2022;73:135–42. 10.1016/j.copbio.2021.07.003. 34392234 10.1016/j.copbio.2021.07.003

[11] Cole JR, Wang Q, Fish JA, Chai B, McGarrell DM, Sun Y, et al. Ribosomal database project: data and tools for high throughput rRNA analysis. Nucleic Acids Res. 2014;42(D1):D633–42. 10.1093/NAR/GKT1244. 24288368 10.1093/nar/gkt1244 PMC3965039

[12] Cook DE, Mesarich CH, Thomma BPHJ. Understanding plant immunity as a surveillance system to detect invasion. Annu Rev Phytopathol. 2015:541–563. 10.1146/ANNUREV-PHYTO-080614-120114. 10.1146/annurev-phyto-080614-120114 26047564

[13] de Jonge R, van Esse HP, Maruthachalam K, Bolton MD, Santhanam P, Saber MK, et al. Tomato immune receptor Ve1 recognizes effector of multiple fungal pathogens uncovered by genome and RNA sequencing. Proc Natl Acad Sci U S A. 2012;109(13):5110–5. 10.1073/pnas.1119623109. 22416119 10.1073/pnas.1119623109 PMC3323992

[14] Du Y, Han X, Tsuda K. Microbiome-mediated plant disease resistance: recent advances and future directions. J Gen Plant Pathol. 2025;91(1):1–17. 10.1007/s10327-024-01204-1.

[15] Dumack K, Feng K, Flues S, Sapp M, Schreiter S, Grosch R, et al. What drives the assembly of plant-associated protist microbiomes? Investigating the effects of crop species, soil type and bacterial microbiomes. Protist. 2022;173:125913. 10.1016/j.protis.2022.125913. 36257252 10.1016/j.protis.2022.125913

[16] Durán P, Thiergart T, Garrido-Oter R, Agler M, Kemen E, Schulze-Lefert P, et al. Microbial interkingdom interactions in roots promote *Arabidopsis* survival. Cell. 2018;175(4):973-983.e14. 10.1016/j.cell.2018.10.020. 30388454 10.1016/j.cell.2018.10.020 PMC6218654

[17] Fierer N. Embracing the unknown: disentangling the complexities of the soil microbiome. Nat Rev Microbiol. 2017;15(10):579–90. 10.1038/nrmicro.2017.87. 28824177 10.1038/nrmicro.2017.87

[18] Fitzpatrick CR, Copeland J, Wang PW, Guttman DS, Kotanen PM, Johnson MTJ. Assembly and ecological function of the root microbiome across angiosperm plant species. Proc Natl Acad Sci U S A. 2018;115(6):E1157–65. 10.1073/pnas.1717617115. 29358405 10.1073/pnas.1717617115 PMC5819437

[19] Fradin EF, Thomma BPHJ. Physiology and molecular aspects of Verticillium wilt diseases caused by *V. dahliae* and *V. albo-atrum*. Mol Plant Pathol. 2006;7(2):71–86. 10.1111/j.1364-3703.2006.00323.x. 20507429 10.1111/j.1364-3703.2006.00323.x

[20] Gómez-Pérez D, Schmid M, Chaudhry V, Hu Y, Velic A, Maček B, et al. Proteins released into the plant apoplast by the obligate parasitic protist *Albugo* selectively repress phyllosphere-associated bacteria. New Phytol. 2023;239(6):2320–34. 10.1111/nph.18995. 37222268 10.1111/nph.18995

[21] Guerreiro MA, Stukenbrock EH. Fungal plant pathogens. Curr Biol. 2025;35(11):R480–4. 10.1016/j.cub.2025.02.046. 40494300 10.1016/j.cub.2025.02.046

[22] Hartemink AE, Sonneveld MPW. Soil maps of The Netherlands. Geoderma. 2013;204:1–9. 10.1016/j.geoderma.2013.03.022.

[23] Hassani MA, Durán P, Hacquard S. Microbial interactions within the plant holobiont. Microbiome. 2018;6(1):58. 10.1186/s40168-018-0445-0. 29587885 10.1186/s40168-018-0445-0 PMC5870681

[24] Jones JDG, Dangl JL. The plant immune system. Nature. 2006;444(7117):323–9. 10.1038/nature05286. 17108957 10.1038/nature05286

[25] Katan J. Diseases caused by soilborne pathogens: biology, management, challenges. J Plant Pathol. 2017;99(2):305–15.

[26] Kettles GJ, Bayon C, Sparks CA, Canning G, Kanyuka K, Rudd JJ. Characterization of an antimicrobial and phytotoxic ribonuclease secreted by the fungal wheat pathogen *Zymoseptoria tritici*. New Phytol. 2018;217(1):320–31. 10.1111/nph.14786. 28895153 10.1111/nph.14786 PMC5724701

[27] Klimes A, Dobinson KF, Thomma BPHJ, Klosterman SJ. Genomics spurs rapid advances in our understanding of the biology of vascular wilt pathogens in the genus *Verticillium*. Annu Rev Phytopathol. 2015;53:181–98. 10.1146/annurev-phyto-080614-120224. 26047557 10.1146/annurev-phyto-080614-120224

[28] Kraege A, Punt W, Doddi A, Zhu J, Schmitz N, Snelders NC, et al. Undermining the cry for help: the phytopathogenic fungus *Verticillium dahliae* secretes an antimicrobial effector protein to undermine host recruitment of antagonistic Pseudomonas bacteria. bioRxiv. 2025. 10.1101/2025.06.09.658588. 10.1111/nph.70686 PMC12676067 41163408

[29] Liu Y, Chen L, Wu G, Feng H, Zhang G, Shen Q, et al. Identification of root-secreted compounds involved in the communication between cucumber, the beneficial *Bacillus amyloliquefaciens*, and the soil-borne pathogen *Fusarium oxysporum*. Mol Plant Microbe Interact. 2017;30(1):53–62. 10.1094/MPMI-07-16-0131-R. 27937752 10.1094/MPMI-07-16-0131-R

[30] Love MI, Huber W, Anders S. Moderated estimation of fold change and dispersion for RNA-seq data with DESeq2. Genome Biol. 2014;15(12):1–21. 10.1186/S13059-014-0550-8/FIGURES/9. 10.1186/s13059-014-0550-8 PMC4302049 25516281

[31] Lundberg DS, Lebeis SL, Herrera Paredes S, Yourstone S, Gehring J, Malfatti S, et al. Defining the core *Arabidopsis thaliana* root microbiome. Nature. 2012;488(7409):86–90. 10.1038/nature11237. 22859206 10.1038/nature11237 PMC4074413

[32] Martin M. Cutadapt removes adapter sequences from high-throughput sequencing reads. EMBnet J. 2011;17(1):10–2. 10.14806/EJ.17.1.200.

[33] McMurdie PJ, Holmes S. phyloseq: an R package for reproducible interactive analysis and graphics of microbiome census data. PLoS ONE. 2013;8(4):e61217. 10.1371/JOURNAL.PONE.0061217. 23630581 10.1371/journal.pone.0061217 PMC3632530

[34] Mendes R, Kruijt M, de Bruijn I, Dekkers E, van der Voort M, Schneider JHM, et al. Deciphering the rhizosphere microbiome for disease-suppressive bacteria. Science. 2011;332(6033):1097–100. 10.1126/science.1203980. 21551032 10.1126/science.1203980

[35] Mesny F, Bauer M, Zhu J, Thomma BPHJ. Meddling with the microbiota: fungal tricks to infect plant hosts. Curr Opin Plant Biol. 2024;82:102622. 10.1016/j.pbi.2024.102622. 39241281 10.1016/j.pbi.2024.102622

[36] Mesny F, Thomma BPHJ. AMAPEC: accurate antimicrobial activity prediction for fungal effector proteins. bioRxiv. 2024. 10.1101/2024.01.04.574150.

[37] Morton JT, Toran L, Edlund A, Metcalf JL, Lauber C, Knight R. Uncovering the horseshoe effect in microbial analyses. mSystems. 2017. 10.1128/msystems.00166-16. 28251186 10.1128/mSystems.00166-16 PMC5320001

[38] Oksanen J, Simpson G, Blanchet F, Kindt R, Legendre P, Minchin P, et al. vegan: community ecology package. 2004. p. https://github.com/vegandevs/vegan.

[39] Ofek-Lalzar M, Sela N, Goldman-Voronov M, Green SJ, Hadar Y, Minz D. Niche and host-associated functional signatures of the root surface microbiome. Nat Commun. 2014;5:4950. 10.1038/ncomms5950. 25232638 10.1038/ncomms5950

[40] Ökmen B, Katzy P, Huang L, Wemhöner R, Doehlemann G. A conserved extracellular Ribo1 with broad-spectrum cytotoxic activity enables smut fungi to compete with host-associated bacteria. New Phytol. 2023;240(5):1976–89. 10.1111/nph.19244. 37680042 10.1111/nph.19244

[41] Pieterse CMJ, Zamioudis C, Berendsen RL, Weller DM, van Wees SCM, Bakker PAHM. Induced systemic resistance by beneficial microbes. Annu Rev Phytopathol. 2014;52:347–75. 10.1146/annurev-phyto-082712-102340. 24906124 10.1146/annurev-phyto-082712-102340

[42] Poppeliers SWM, Sánchez-Gil JJ, López JL, Dutilh BE, Pieterse CMJ, Jonge R de. High-resolution quantification of the rhizosphere effect along a soil-to-root gradient shows selection-driven convergence of rhizosphere microbiomes. bioRxiv. 2024;2024.06.21.600027. 10.1101/2024.06.21.600027.

[43] Raaijmakers JM, Weller DM. Natural plant protection by 2,4-Diacetylphloroglucinol-producing Pseudomonas spp. in take-all decline soils. Mol Plant Microbe Interact. 1998;11(2):144–52.

[44] Schneider CA, Rasband WS, Eliceiri KW. NIH Image to ImageJ: 25 years of image analysis. Nat Methods. 2012;9(7):671–5. 10.1038/nmeth.2089. 10.1038/nmeth.2089 PMC5554542 22930834

[45] Simonin M, Dasilva C, Terzi V, Ngonkeu ELM, Diouf D, Kane A, et al. Influence of plant genotype and soil on the wheat rhizosphere microbiome: evidences for a core microbiome across eight African and European soils. FEMS Microbiol Ecol. 2020;96(6). 10.1093/femsec/fiaa067. 10.1093/femsec/fiaa067 32275297

[46] Singh BK, Jiang G, Wei Z, Sáez-Sandino T, Gao M, Liu H, et al. Plant pathogens, microbiomes, and soil health. Trends Microbiol. 2025;33(8):887–902. 10.1016/j.tim.2025.03.013. 40274492 10.1016/j.tim.2025.03.013

[47] Snelders NC, Boshoven JC, Song Y, Schmitz N, Fiorin GL, Rovenich H, et al. A highly polymorphic effector protein promotes fungal virulence through suppression of plant-associated Actinobacteria. New Phytol. 2023;237(3):944–58. 10.1111/nph.18576. 36300791 10.1111/nph.18576

[48] Snelders NC, Petti GC, van den Berg GCM, Seidl MF, Thomma BPHJ. An ancient antimicrobial protein co-opted by a fungal plant pathogen for in planta mycobiome manipulation. Proc Natl Acad Sci USA. 2021;118(49):e2110968118. 10.1073/PNAS.2110968118/-/DCSUPPLEMENTAL. 34853168 10.1073/pnas.2110968118 PMC8670511

[49] Snelders NC, Rovenich H, Petti GC, Rocafort M, van den Berg GCM, Vorholt JA, et al. Microbiome manipulation by a soil-borne fungal plant pathogen using effector proteins. Nat Plants. 2020;6(11):1365–74. 10.1038/s41477-020-00799-5. 33139860 10.1038/s41477-020-00799-5

[50] Snelders NC, Rovenich H, Thomma BPHJ. Microbiota manipulation through the secretion of effector proteins is fundamental to the wealth of lifestyles in the fungal kingdom. FEMS Microbiol Rev. 2022;46(5). 10.1093/femsre/fuac022. 10.1093/femsre/fuac022 PMC9438471 35604874

[51] Sokol NW, Slessarev E, Marschmann GL, Nicolas A, Blazewicz SJ, Brodie EL, et al. Life and death in the soil microbiome: how ecological processes influence biogeochemistry. Nat Rev Microbiol. 2022;20(7):415–30. 10.1038/s41579-022-00695-z. 35228712 10.1038/s41579-022-00695-z

[52] Spooren J, van Bentum S, Thomashow LS, Pieterse CMJ, Weller DM, Berendsen RL. Plant-driven assembly of disease-suppressive soil microbiomes. Annu Rev Phytopathol. 2024;62(1):1–30. 10.1146/annurev-phyto-021622-100127. 38857541 10.1146/annurev-phyto-021622-100127

[53] Thiergart T, Durán P, Ellis T, Vannier N, Garrido-Oter R, Kemen E, et al. Root microbiota assembly and adaptive differentiation among European Arabidopsis populations. Nat Ecol Evol. 2020;4(1):122–31. 10.1038/s41559-019-1063-3. 31900452 10.1038/s41559-019-1063-3

[54] Tkacz A, Bestion E, Bo Z, Hortala M, Poole PS. Influence of plant fraction, soil, and plant species on microbiota: a multikingdom comparison. mBio. 2020;11(1). 10.1128/mBio.02785-19. 10.1128/mBio.02785-19 PMC7002342 32019791

[55] Trivedi P, Leach JE, Tringe SG, Sa T, Singh BK. Plant-microbiome interactions: from community assembly to plant health. Nat Rev Microbiol. 2020;18(11):607–21. 10.1038/s41579-020-0412-1. 32788714 10.1038/s41579-020-0412-1

[56] van der Heijden MGA, Hartmann M. Networking in the plant microbiome. PLoS Biol. 2016;14(2):e1002378. 10.1371/journal.pbio.1002378. 26871440 10.1371/journal.pbio.1002378 PMC4752285

[57] Vandenkoornhuyse P, Quaiser A, Duhamel M, Le Van A, Dufresne A. The importance of the microbiome of the plant holobiont. New Phytol. 2015;206(4):1196–206. 10.1111/nph.13312. 25655016 10.1111/nph.13312

[58] Wagner MR, Lundberg DS, Del Rio TG, Tringe SG, Dangl JL, Mitchell-Olds T. Host genotype and age shape the leaf and root microbiomes of a wild perennial plant. Nat Commun. 2016;7:12151. 10.1038/ncomms12151. 27402057 10.1038/ncomms12151 PMC4945892

[59] Walters WA, Jin Z, Youngblut N, Wallace JG, Sutter J, Zhang W, et al. Large-scale replicated field study of maize rhizosphere identifies heritable microbes. Proc Natl Acad Sci U S A. 2018;115(28):7368–73. 10.1073/pnas.1800918115. 29941552 10.1073/pnas.1800918115 PMC6048482

